# Nitrogen fixation associated with two cohabiting moss species expresses different patterns under Cu and Zn contamination

**DOI:** 10.1007/s11356-023-28404-0

**Published:** 2023-07-01

**Authors:** Toke Due Sjøgren, Yinliu Wang, Kathrin Rousk

**Affiliations:** 1 Department of Biology, Center for Volatile Interactions (VOLT), University of Copenhagen, Universitetsparken 15, DK-2100 Copenhagen, Denmark; 2 State Key Laboratory of Vegetation and Environmental Change, Institute of Botany, the Chinese Academy of Sciences, Beijing, 100093 China

**Keywords:** Bryophytes, Cyanobacteria, Heavy metal pollution, Nitrogen fixation, *Sphagnum*

## Abstract

Nitrogen (N_2_) fixation by moss-associated cyanobacteria is an important N source in pristine ecosystems. Previous studies have shown that moss-associated N_2_ fixation is sensitive to anthropogenic N pollution. However, we still lack understanding of the effects of other factors derived from anthropogenic sources, such as heavy metal pollution on N_2_ fixation. To test this, we collected two dominant mosses (*Pleurozium schreberi* and *Spaghnum palustre*) from a temperate bog in Denmark and assessed their N_2_ fixation responses to simulated heavy metal pollution by adding 5 levels (plus a control) of copper (Cu, 0–0.05 mg g dw^−1^) and zinc (Zn, 0–0.1 mg g dw^−1^). Metal concentrations in both mosses increased linearly with Cu and Zn addition, but N_2_ fixation activity associated with *S. palustre* was to a greater extent negatively affected by both Cu and Zn additions than that associated with *P. schreberi*. Copper additions even promoted N_2_ fixation in *P. schreberi*. Hence, the heavy metal sensitivity of N_2_-fixing cyanobacteria is dependent on the host moss-species, and the vulnerability of ecosystems towards heavy metal pollution could vary depending on the dominant moss species.

**Supplementary Information:**

The online version contains supplementary material available at 10.1007/s11356-023-28404-0.

## Introduction

Mosses are ubiquitous cryptogamic plants that can cover between 70 and 100% of the ground in forest ecosystems (Van Cleve et al. 1986; Rousk et al. 2013), performing numerous ecological roles, such as regulating soil moisture and temperature (Gornall et al. 2007). Moreover, mosses can establish associations with nitrogen (N_2_)-fixing cyanobacteria that can provide new N to N-limited ecosystems, indicating that moss-associated N_2_ fixation is invaluable for plant productivity in unpolluted areas (DeLuca et al. 2002; Rousk et al. 2013; Alvarenga and Rousk 2022).

Moss-associated N_2_-fixing ability has been investigated thoroughly in relation to increased N deposition and N availability and all studies show fast, inhibitory effects of N on N_2_ fixation in mosses (Ackermann et al. 2012; Goth et al. 2019; Rousk et al. 2013). However, the effect of other pollutants, such as heavy metals, on N_2_ fixation associated with mosses is still relatively understudied, especially the effects of individual heavy metals remain largely unexplored—even though negative effects are to be expected.

Prolonged exposure as well as the quantity of heavy metals negatively impact N_2_ fixation in both symbiotic diazotrophs (N_2_-fixing organism) in soil and free-living diazotrophs in soil and moss (Oliveira and Pampulha 2006). However, Goth et al. (2019) found a lack of inhibition on moss-associated N_2_ fixation when examining heavy metal deposition of Cu, Zn, Fe, and Pb along a distance gradient away from a railway in an otherwise pristine subarctic tundra system—even though moss-Fe content was 50× higher than the national average in the same country. Akther and Rousk (2019) also detected that even though heavy metal input might impact mosses, the N_2_ fixation activity of colonizing cyanobacteria was not inhibited and, in some cases, promoted by iron availability along a distance gradient away from a metal smelter in Northern Sweden. Again, the metal content of mosses was several folds higher close to the metal smelter compared to further away. These findings indicate that moss associated N_2_ fixation is less affected than previously assumed and the response is dependent on the specific heavy metal.

Besides being components in automobile transportation and industrial emissions (Jiang et al. 2018), copper (Cu) and zinc (Zn) are micronutrients needed in small quantities as constituents of several essential proteins in plants (Hänsch and Mendel 2009). Accumulation beyond a certain concentration, however, will induce toxicity and disrupt metabolic and photosynthetic processes, resulting in reduced plant development (McCauley et al. 2009). Most of the available data focusing on heavy metal pollution effects on mosses are related to their biomonitoring functions (Jiang et al. 2018; Kłos et al. 2018; Mao et al. 2022). However, there is a noticeable lack of studies investigating heavy metal effects on moss-associated N_2_ fixation. Furthermore, most of the very few related studies focused on epiphytic cyanobacteria in association with the common feather moss species *Pleurozium schreberi* (Akther and Rousk 2019; Goth et al. 2019; Rousk et al. 2013). While other mosses are neglected, such as *Spaghnum* mosses, where the associated diazotrophs are mainly hosted within dead cells, and which are key ecosystem components in peatlands and bogs that are large carbon sinks (van den Elzen et al. 2020). In order to fill these knowledge gaps, we assessed moss-related cyanobacterial N_2_ fixation activity of *P. schreberi* and *Sphagnum palustre* in response to the application of different concentrations of Cu and Zn over a period of 3 weeks.

We hypothesized that (H1) N_2_ fixation declines with increasing heavy metal additions similarly for both Zn and Cu as additions were adjusted for naturally occurring heavy metal content in the mosses and the toxicity of the metals. (H2) N_2_ fixation associated with *P. schreberi* is more inhibited by Cu and Zn additions than N_2_ fixation associated with *S. palustre* due to the different cyanobacterial symbiont locations—epiphytically for *P. schreberi* and endophytically for *S. palustre*. (H3) the inhibition of N_2_ fixation is strongest directly after the addition of heavy metals.

## Materials and methods

### Sampling sites and Cu and Zn additions

The sampling site is situated in the relatively pristine environment “Bøllemosen” (55°49′31″N, 12°34′26″E) north of Copenhagen, Denmark, and is characterized as an earlier high moor that has become a raised bog adjacent to a dystrophic lake due to peat excavation (Ministry of the Environment, Denmark n.d.). The annual mean temperature and precipitation in Bøllemosen is 9.46 °C and 654 mm, respectively. Precipitation is evenly distributed across the year (“World Bank Climate Change Knowledge Portal,” https://climateknowledgeportal.worldbank.org/country/denmark/climate-data-historical). The pH of the bog area where we collected the mosses from is 3.8 ± 0.1 (unpublished data). Moss samples were collected in February 2022 from 4 random locations with approximately 3–4 m between each sampling plot. One full plastic bag of moss was collected at each sampling site (= replicates) and transported back to the laboratory to be weighed into 20 ml glass vials: 5 g fresh weight (fw) of *Pleurozium schreberi* (Brid.) Mitt. and 5.7 g fw of *Sphagnum palustre* (L.) corresponding to 0.2 g dry weight.

We chose Cu and Zn for the additions as they are essential for plant growth but induce toxicity in high concentrations (Sabovljević et al. 2018). The levels of addition were taken from previous studies that reported heavy metal content in *P. schreberi* in unpolluted habitats (Akther and Rousk 2019; Goth et al. 2019). Following these studies, we added 0 (control), 0.02, 0.04, 0.06, 0.08, and 0.1 mg g dw^−1^ for Zn, and 0, 0.01, 0.02, 0.03, 0.04, and 0.05 mg g dw^−1^ for Cu. The solutions were created from a stock solution of respectively 20 mg/L ZnSO_4_ and 10 mg/L CuSO_4_, diluted with double distilled water. The samples were inoculated once with 1 ml of the corresponding heavy metal concentration and placed in a growth chamber with a constant temperature of 10 °C and 12-h day/night cycles with photosynthetic active radiation (PAR) of 229 μmol m^−2^ s^−1^.

### Nitrogen fixation assessed with the acetylene reduction assay

Nitrogen fixation was assessed using the acetylene reduction assay (ARA), which quantifies the activity of the nitrogenase enzyme that reduces N_2_ as well as acetylene. Acetylene is reduced to ethylene gas by nitrogenase, which can be easily measured using gas chromatography. Prior to the ARAs, the 20 ml glass vials containing the moss samples were capped with airtight rubber septa and 10% (2 ml) of the headspace was replaced with acetylene gas. Samples were incubated with acetylene for ca. 24 h in the growth chamber with the same conditions as described above. Samples, standards, and acetylene blanks (acetylene-only to account for any ethylene residue in the acetylene gas, which was subtracted from the ethylene produced in the samples) were then run at 10 psi, 60 °C oven temperature on a gas chromatograph coupled to a headspace sampler, fitted with a FID (Agilent 8890, Agilent Technologies, Santa Clara, CA, USA). The ARA was conducted four times (1, 7, 14, and 21 days) after the addition of heavy metals. Samples were oven dried (24 h at 70 °C) immediately after the last measurements for heavy metal analyses (below).

### Heavy metal analyses

The concentration of heavy metal in each sample was analyzed using atomic absorption spectroscopy (AAS) (27th April–4th May 2022), following the last acetylene reduction measurements. For this, the dried moss samples were cut with scissors and digested with 65% HNO_3_ in a MARS 6 microwave. The flame setting of the Perkin Elmer Atomic Absorption Spectrometer PinAAcle 900T was used to measure the specific heavy metal content in each sample. The amount of Cu in the control samples was not able to be accurately determined as the concentrations were close to the detection limit; therefore, we set the Cu concentration in controls as 0.

### Data analysis

Links between heavy metal additions and moss heavy metal concentration, between heavy metal addition and response ratio of acetylene reduction (RR) over time were assessed with linear regression analysis. Differences in heavy metal concentrations and RR between the moss species and heavy metal types were tested using the two-way ANOVA, followed by Duncan’s test. Acetylene reduction RR at different heavy metal addition rates was calculated as acetylene reduced at different rates and types of heavy metal addition divided by the rates in control samples (Wang et al. 2022). All data was log transformed to conform with the assumptions of normality and homoscedasticity before analyses. All data was analyzed using R 4.2.1 (R Core Team 2022).

## Results

### Heavy metal content and concentration

**Fig. 1:**
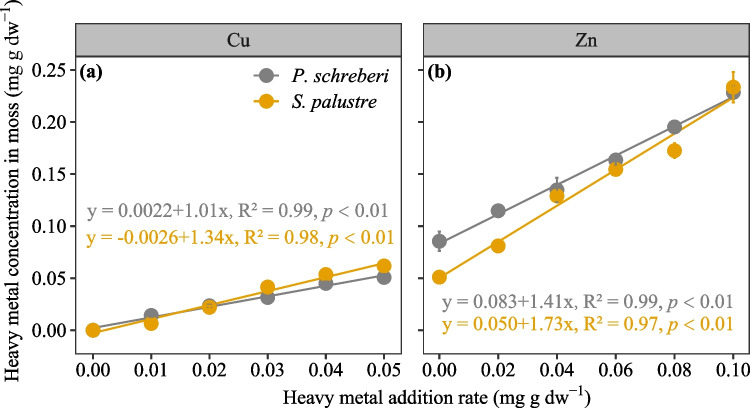
Relationship between heavy metal addition rate (mg g dw^−1^) and heavy metal concentration (mg g dw^−1^) in two moss species (*P. schreberi* in grey, and *S. palustre* in yellow) collected from a temperature bog and exposed to Cn and Zn additions in the laboratory. Given are mean values ± SE (*n* = 4)

Cu content increased with addition rates for *P. schreberi* (R^2^ = 0.99, *p* < 0.01; Fig. 1a) and *S. palustre* (R^2^ = 0.97, *p* < 0.01). As did the content of Zn in *P. schreberi* (R^2^ = 0.99, *p* < 0.01; Fig. 1b) and *S. palustre* (R^2^ = 0.97, *p* < 0.01). Overall, there was no significant difference in Cu or Zn content between the two moss species after heavy metal addition (Fig. S1). Levels of Zn in both *P. schreberi* and *S. palustre* were higher than the added concentrations, and the controls (0 Zn additions) were above 0, indicating previous Zn pollution or exposure at the sampling site.

### Nitrogen fixation activity

**Fig. 2:**
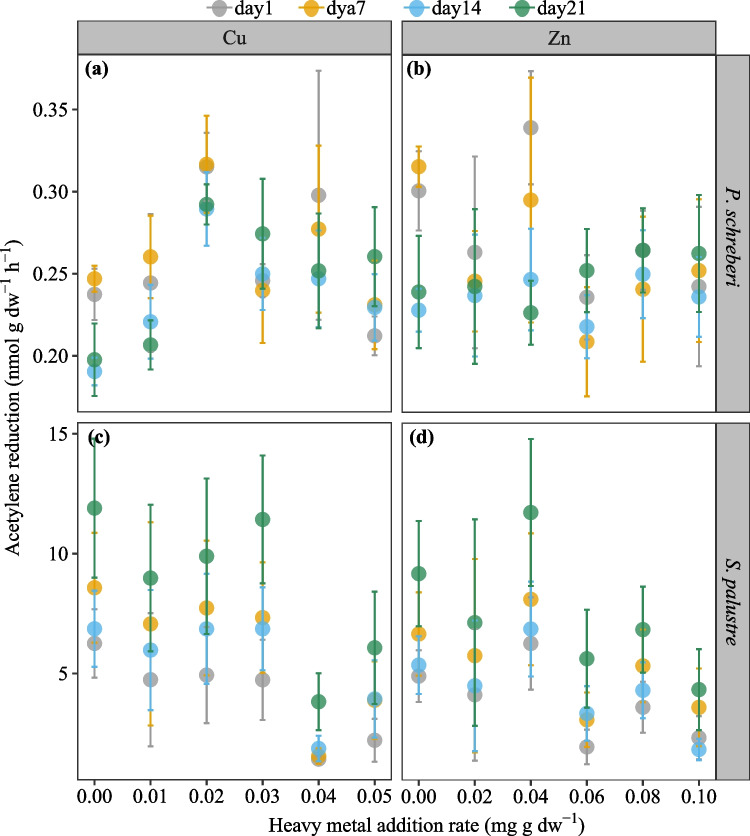
Nitrogen fixation, measured as acetylene reduction rates, in *P. schreberi* (top panels) and *S. palustre* (lower panels) measured 1 (day1), 7 (day7), 14 (day14), and 21 (day21) days after additions of Cu **a**, **c** and Zn **b**, **d** at different doses. Given are mean values ± SE (*n* = 4)

**Fig. 3:**
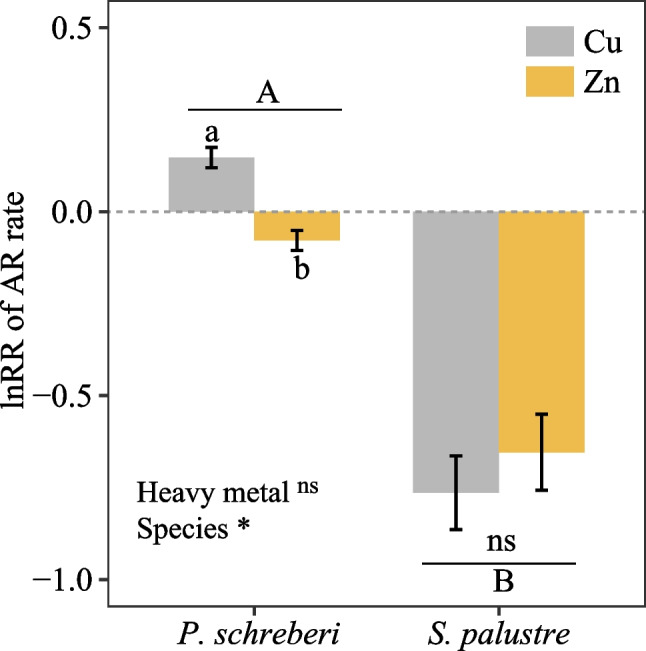
Response ratio (log-transformed, lnRR) of acetylene reduction of *P. schreberi* and *S. palustre* towards Cu and Zn additions across all doses and times. Given are mean values ± SE (*n* = 80). Different lower-case and upper-case letters denote significant differences (*p* < 0.05, Duncan’s tests) between heavy metal types and moss species, respectively. ns indicates no significant difference (*p* > 0.05) and * indicates significant effects of the treatments (*p* < 0.05) based on two-way ANOVA results

Overall, both Cu and Zn addition had no significant effects on the amount of acetylene reduced by cyanobacteria associated with *P. schreberi* (Figs. 2a, b, S2a), but acetylene reduction rates after Cu additions were slightly higher than after Zn additions (*p* < 0.05; Figs. 3a, S2a, Table S1). Also, nitrogenase activity increased by ca. a third when doubling Cu levels from 0.01 to 0.02 mg g dw^−1^ 3 weeks after the additions (Fig. 2a), while nitrogenase activity decreased by the same magnitude over time in, e.g., the 0.04 mg g dw^−1^ Zn additions (Fig. 2b). Acetylene reduction rates of *S. palustre* halved when Cu and Zn addition rates were above 0.03 mg g dw^−1^ for Cu and 0.04 mg g dw^−1^ for Zn (Fig. 2c, d). Acetylene reduction of *P. schreberi-*associated cyanobacteria was positively affected by Cu and inhibited by Zn (Fig. 3a), as the log-transformed response ratio (lnRR) of acetylene reduction for *P. schreberi* was always > 0 under Cu addition and < 0 for Zn addition (Fig. 3a). In contrast, there was no significant difference in acetylene reduction in *S. palustre* between Cu and Zn additions, which both showed similar inhibiting effects (Fig. 3b).

### Nitrogen fixation activity over time

**Fig. 4:**
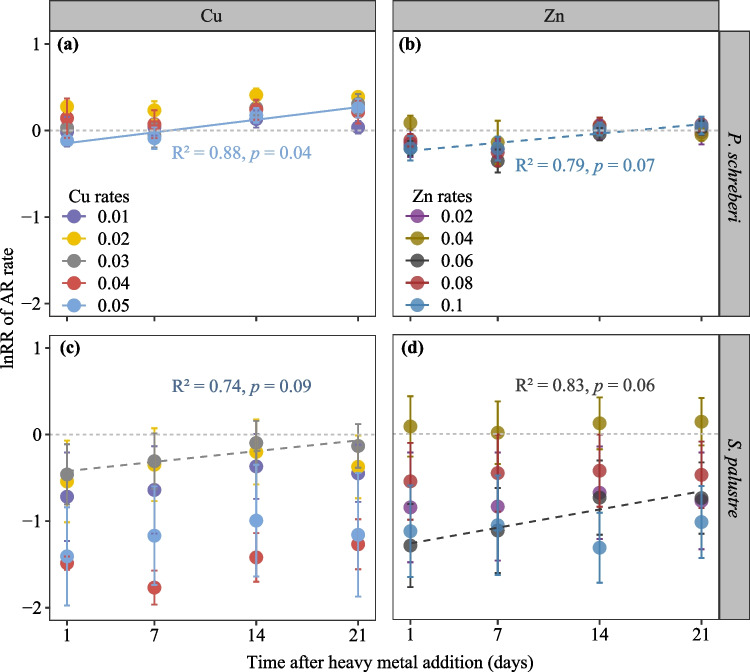
Relationship between log-transformed acetylene reduction response ratios (lnRR) of *P. schreberi* and *S. palustre* after Cu and Zn additions over time. Given are mean values ± SE (*n* = 4). Different colour represents different heavy metal addition rates. lnRR with error bars not overlapping with lnRR = 0 indicates that treatment significantly increased (where lnRR > 0) or inhibited (where lnRR < 0) acetylene reduction comparing to the control treatment. Solid lines indicate significant levels at *p* ≤ 0.05, and dotted lines indicate significant levels at 0.05 < *p* < 0.1

The response ratio of acetylene reduction (lnRR) showed an increasing trend with time in both moss species (Fig. 4). The lnRR of *P. schreberi* under all treatments was above or overlapped with 0 after 21 days (Fig. 4a, b), and a significant increasing pattern over time was found with Cu addition at 0.05 mg g dw^−1^ (R^2^ = 0.88, *p* = 0.04; Fig. 4a). In contrast, the response ratios of *S. palustre* were below or overlapped with 0 after 3 weeks of the addition for both metals (Fig. 4c, d). Though the increasing patterns of lnRR over time were only marginal significant, acetylene reduction rates were higher 21 days after the addition compared to 1 day after the additions for both metals in *S. palustre* (Fig. S2b).

## Discussion

### Heavy metal additions

Our first hypothesis (H1), that N_2_ fixation declines with increasing heavy metal additions equally for both Zn and Cu, was only partly confirmed as we found a positive effect of Cu and a negative effect of Zn on N_2_ fixation activity associated with *P. schreberi* (Figs. 3, S2a). However, the hypothesis holds true for *S. palustre*, where associated N_2_ fixation was similarly inhibited by both heavy metals. The different responses towards the two heavy metals in *P. schreberi* are most likely explained by the differing levels of addition as 0.05 mg^−1^ g dw^−1^ Cu could be insufficient to induce inhibition in *P. schreberi* while 0.1 mg^−1^ g dw^−1^ Zn was sufficient*.* Here, Cu could have been stored in cell walls (Bernal et al. 2006), or even immobilized within the cell rendering it ineffective. Indeed, inputs of various heavy metals have been shown before to not affect N_2_ fixation in *P. schreberi* (Ackermann et al. 2012; Akther and Rousk 2019). However, another explanation could be the selective transport and uptake of different heavy metals (Stanković et al. 2018) resulting in a varied accumulation of Cu and Zn in *P. schreberi*.

We cannot exclude confounding effects of our experimental design that may have impacted our results. Using sulfate in our additions could have impaired the performance of cyanobacteria (and moss)—yet, given that we detected promotion (by CuSO4 additions), and inhibition (by ZnSO4 additions) of N_2_ fixation by adding sulfate-containing compounds, the observed effects are likely due to the different metals, and not the sulfate. Metal additions can also lower pH levels (e.g., Rousk et al. 2012), which can inhibit moss-associated N_2_ fixation (Wang et al. 2022). Nonetheless, given that nitrogenase activity slightly increased with time, any potential negative effects of lowered pH seem to have been short-lived.

### Species differences in the sensitivity of nitrogen fixation towards heavy metal additions

Our results cannot support our second hypothesis (H2) that nitrogenase activity associated with *P. schreberi* will show a greater inhibition than in *S. palustre* due to different localization of associated cyanobacteria. On the contrary, N_2_ fixation associated with *S. palustre* seems to be more sensitive to heavy metal input compared to *P. schreberi* (Figs. 3 and 4). Several reasons may explain the differences in sensitivity between the moss species towards Zn and Cu pollution. Firstly, for *S. palustre*, the dead hyaline cells housing the cyanobacteria could allow greater heavy metal accumulation than the living *P. schreberi* cells occupied by epiphytic cyanobacteria, where heavy metals may easily be washed off. Secondly, the cyanobacteria associated with *P. schreberi* may be spared the detrimental effects of heavy metal accumulation as *P. schreberi* might take up most of the added heavy metal, either as part of cell metabolism or immobilized outside the cell, within the cell walls. Indeed, no negative effect of high heavy metal input on N_2_ fixation associated with the same moss species, *P. schreberi*, has been found in field assessments (e.g., Ackermann et al. 2012; Goth et al. 2019; Akther and Rousk 2019). This indicates that this particular moss species is either efficient in taking up heavy metals, thereby preventing exposure of associated cyanobacteria to the pollutants, or thirdly, the bacteria colonizing the two mosses may be different, and the ones associated with *P. schreberi* may be less susceptible to the pollution. Indeed, moss-associated bacterial communities do seem to be specific to different moss hosts (Alvarenga and Rousk 2022). Finally, and fourthly, moss species have different intracellular mechanisms, as reported by (Hänsch and Mendel 2009). For instance, *P. schreberi* could have higher production and concentration of antioxidants like glutathione and sequestering molecules like metallothioneins mitigating the deleterious effects of Zn and Cu. Additionally other explanations could be the selective uptake and transport of heavy metals across the protoplast (Stanković et al. 2018). Nonetheless, our results highlight the need for further studies to identify the mechanisms that allow some N_2_-fixing bacteria to be less affected by heavy metal pollution than others, and this sensitivity seems to depend on the moss host.

### Decreasing effects of heavy metal pollution on nitrogen fixation over time

Though N_2_ fixation rates after heavy metal additions in *S. palustre* were still lower than that in the controls, N_2_ fixation rates tended to increase over time, which was in line with the third hypothesis (H3) that the inhibition of N_2_ fixation would be strongest directly after heavy metal addition. While adaptation of cyanobacteria to heavy metal stress has been found previously (Tandeau de Marsac and Houmard 1993), the duration of our experiment here (3 weeks) was likely too short to detect potential acclimation or adaptation. Another possible explanation for this observed increase in N_2_ fixation activity over time could be an increased production of sequestration molecules by the moss that mitigate the harmful effects of the heavy metals, or incorporation of the micronutrients in enzymes and structural proteins by the mosses (Hänsch and Mendel 2009) that would lower the exposure to the colonizing cyanobacteria. Hence, it seems that recovery of N_2_ fixation after heavy metal exposure is possible, but it may take several weeks (> 3 weeks) before full recovery is achieved.

## Conclusions

Our results show that moss-associated, N_2_-fixing cyanobacteria are differently affected by heavy metal accumulation for *S. palustre* vs. *P. schreberi*. Nitrogen fixation associated with *S. palustre* was more negatively affected by the here studied heavy metals, most likely due to the different locations of the bacteria associated with the mosses, or differences in cellular heavy metal mitigation mechanisms. Additionally, Cu induced the largest inhibition of N_2_ fixation in *S. palustre*, but lead to a positive response of N_2_ fixation in *P. schreberi.* Furthermore, the effects of Cu and Zn additions decreased over time, and N_2_ fixation even increased with time after exposure, indicating sequestration of heavy metals by the moss host—via higher production of sequestering molecules or incorporation into other molecules or structures such as the vacuole. Several aspects of the effects of heavy metals on moss-associated cyanobacteria are still to be uncovered. For instance, identifying the mechanisms for the here observed differences in N_2_ fixation response between *P. schreberi* and *S. palustre* is worth pursuing. As N_2_ fixation activity was at least 10 times higher in *S. palustre* compared to *P. schreberi*, the sensitivity of *Sphagnum*-associated N_2_ fixation has broad implications as heavy metal pollution will likely lower N input via the N_2_ fixation pathway in systems such as bogs that are dominated by *Sphagnum* mosses. As bogs store large amounts of carbon (C), this decreased N input that supports plant growth may also lower the potential for C sequestration.

Intra-species comparisons of the sensitivity of N_2_ fixation in mosses from polluted vs. pristine habitats would allow to assess adaptation mechanisms of cyanobacteria towards heavy metal pollution. Lastly, extending the moss species comparison to other heavy metals could reveal metal-specific adaptions of N_2_-fixing cyanobacteria.

## Supplementary Information

ESM 1 (DOCX 159 kb)

ESM 2 (XLSX 14 kb)

## Data Availability

Data are available from the authors upon request.
