# Supplementary material for: Nitrogen fixation associated with two cohabiting moss species expresses different patterns under Cu and Zn contamination
Source: Environ Sci Pollut Res Int. 2023 Jul 1;30(36):85701–7. doi: 10.1007/s11356-023-28404-0 (PMC10404191; doi:10.1007/s11356-023-28404-0)
Supplement: Supplementary file 1 — (DOCX 159 kb) [file 11356_2023_28404_MOESM1_ESM.docx]

**Supplement**

**Fig. S1.** Heavy metal concentration of *P. schreberi* and *S. palustre* towards Cu and Zn additions across all doses. Given are mean values ± SE (n = 24). Ns indicates no significant difference (*p* > 0.05) between the moss species based on t-test results.


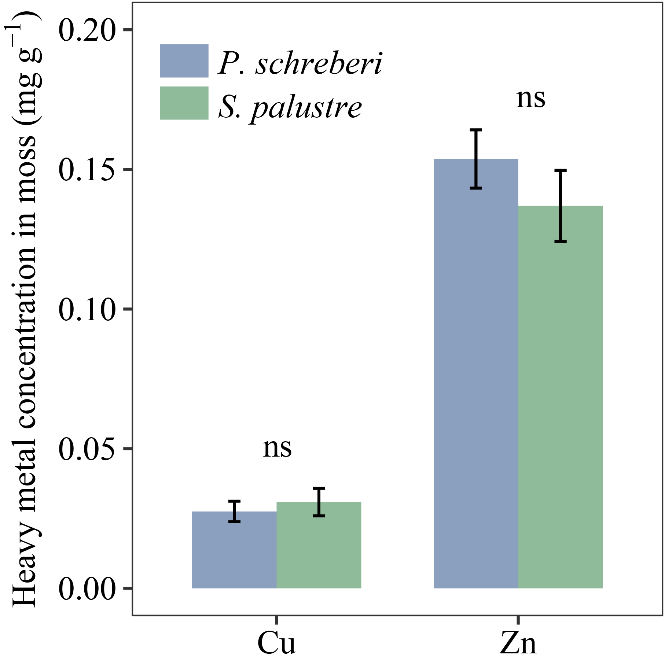


**Fig. S2.** Average AR rates at different times after Cu and Zn additions across all addition rates. Given are mean values ± SE (n = 24). ”ns" indicates insignificant differences (*p* > 0.05) and * indicates significant effects of the treatments (*p* < 0.05) based on two-way ANOVAs results.


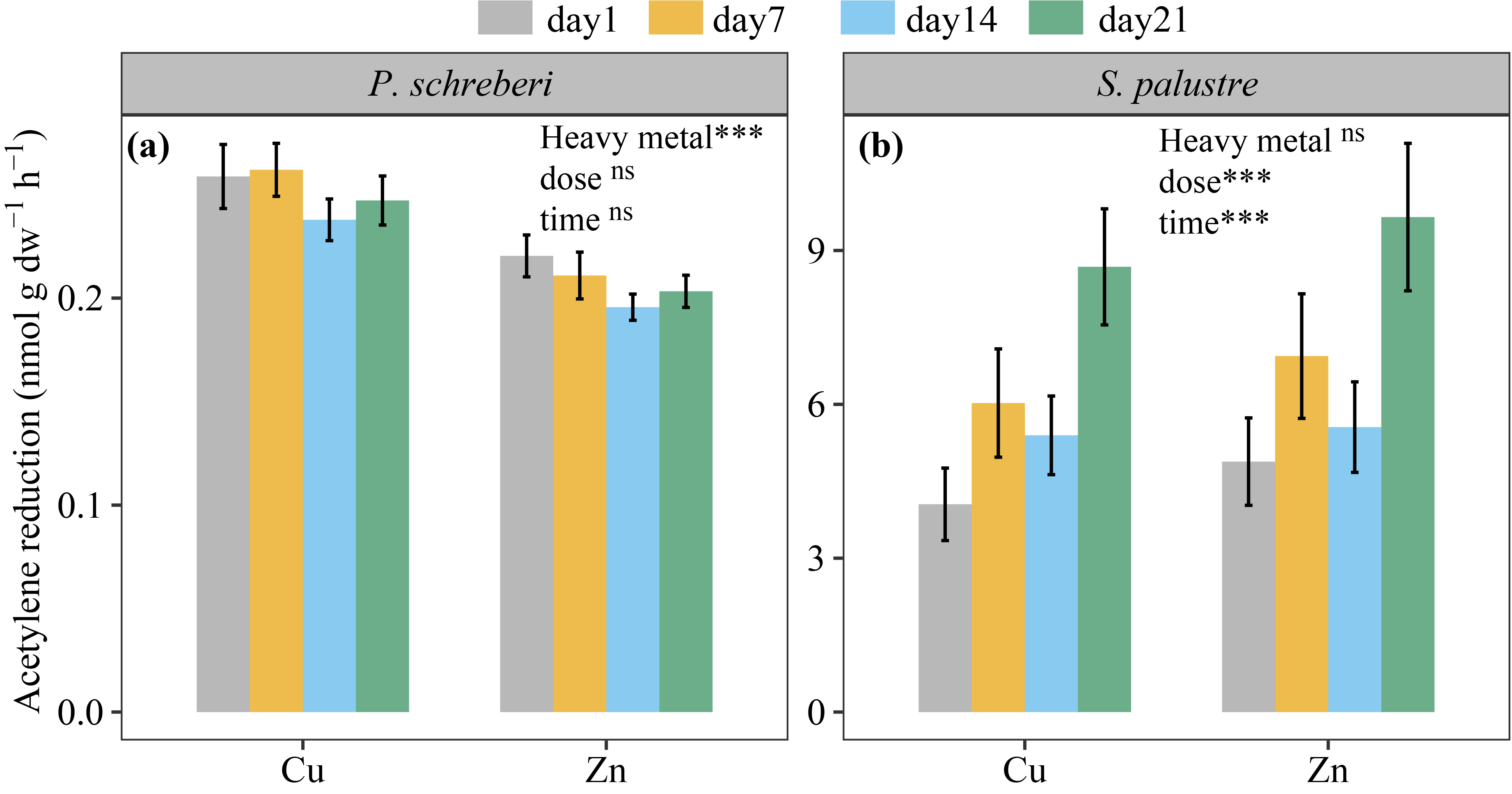


**Table S1.** Ethylene production (nmol g dw^-1^ h^-1^) as a measure of nitrogen fixation in two moss species (*Pleurozium schreberi* and *Sphagnum palustre*) exposed to Zn and Cu additions. Ethylene production was measured at different times after the metal additions. N=4. Supplied as a separate file.
